# Documentation of prenatal contraceptive counseling and fulfillment of permanent contraception: a retrospective cohort study

**DOI:** 10.1186/s12978-024-01752-x

**Published:** 2024-02-14

**Authors:** Ambika V. Viswanathan, Kristen A. Berg, Brooke W. Bullington, Emily S. Miller, Margaret Boozer, Tania Serna, Jennifer L. Bailit, Kavita Shah Arora

**Affiliations:** 1 Department of Obstetrics and Gynecology, University of North Carolina, Chapel Hill, NC 27516 USA; 2 Center for Health Care Research and Policy, Case Western Reserve University, Cleveland, OH 44106 USA; 3 Department of Epidemiology, Gillings School of Global Public Health, University of North Carolina, Chapel Hill, NC 27516 USA; 4 Department of Obstetrics and Gynecology, Division of Maternal Fetal Medicine, Alpert Medical School of Brown University, Providence, RI 02903 USA; 5 Department of Obstetrics and Gynecology, University of Alabama at Birmingham, Birmingham, AL 35233 USA; 6 Department of Obstetrics, Gynecology and Reproductive Sciences, University of California, San Francisco, San Francisco, CA 94143 USA; 7 Department of Obstetrics and Gynecology, MetroHealth Medical Center-Case Western Reserve University, Cleveland, OH 44016 USA

**Keywords:** Prenatal care, Permanent contraception, Fulfillment, Contraception

## Abstract

**Background:**

Barriers exist for the provision of surgery for permanent contraception in the postpartum period. Prenatal counseling has been associated with increased rates of fulfillment of desired postpartum contraception in general, although it is unclear if there is impact on permanent contraception specifically. Thus, we aimed to investigate the association between initial timing for prenatal documentation of a contraceptive plan for permanent contraception and fulfillment of postpartum contraception for those receiving counseling.

**Methods:**

This is a planned secondary analysis of a multi-site cohort study of patients with documented desire for permanent contraception at the time of delivery at four hospitals located in Alabama, California, Illinois, and Ohio over a two-year study period. Our primary exposure was initial timing of documented plan for contraception (first, second, or third trimester, or during delivery hospitalization). We used univariate and multivariable logistic regression to analyze fulfillment of permanent contraception before hospital discharge, within 42 days of delivery, and within 365 days of delivery between patients with a documented plan for permanent contraception in the first or second trimester compared to the third trimester. Covariates included insurance status, age, parity, gestational age, mode of delivery, adequacy of prenatal care, race, ethnicity, marital status, and body mass index.

**Results:**

Of the 3103 patients with a documented expressed desire for permanent contraception at the time of delivery, 2083 (69.1%) had a documented plan for postpartum permanent contraception prenatally. After adjusting for covariates, patients with initial documented plan for permanent contraception in the first or second trimester had a higher odds of fulfillment by discharge (aOR 1.57, 95% C.I 1.24–2.00), 42 days (aOR 1.51, 95% C.I 1.20–1.91), and 365 days (aOR 1.40, 95% C.I 1.11–1.75), compared to patients who had their first documented plan in the third trimester.

**Conclusions:**

Patients who had a documented prenatal plan for permanent contraception in trimester one and two experienced higher likelihood of permanent contraception fulfillment compared to those with documentation in trimester three. Given the barriers to accessing permanent contraception, it is imperative that comprehensive, patient-centered counseling and documentation regarding future reproductive goals begin early prenatally.

## Plain language summary

Permanent contraception is a highly desired form of postpartum contraception in the United States, however there are several barriers to accessing it. In this paper, we investigate whether the timing of when a patient has a documented plan for postpartum contraception has an impact on if they achieve postpartum contraception. This is a cohort study from four hospitals in Illinois, Ohio, California, and Alabama for patients with a desire for postpartum permanent contraception documented in their medical record. We specifically investigated the trimester (first, second, or third) where a patient had a plan for permanent contraception first documented. We then used univariate and multivariate models to determine the relationship between the timing of a plan for permanent contraception and if a patient achieved the procedure at three time-points: hospital discharge, 42-days, and 365-days. Our findings showed that of the 3103 patients in our cohort, only 69.1% of them had a documented plan for postpartum contraception at any point before going to the hospital for their delivery admission. We additionally found that patients who had a documented plan for permanent contraception in the first or second trimester had a higher odds of receiving their postpartum contraception procedure compared to people who had their first documented plan in the third trimester. This showed us the importance of earlier counseling regarding contraception for pregnant patients. There are many barriers to accessing postpartum contraception, so having patient focused counseling about future goals around reproductive health early on in pregnancy is critical.

## Background

While postpartum contraceptive counseling remains an integral component of pregnancy care, the ideal timing and implementation of contraceptive counseling both before and after delivery remains unclear [1]. Prenatal counseling for postpartum contraception is associated with increased postpartum use of contraception in general and is recommended by the American College of Obstetricians and Gynecologists [2–8]. Yet, prenatal contraceptive counseling is not always provided [9]. Potential reasons for this include competing clinical needs for individual patients during time-limited outpatient prenatal office visits, patient and clinician assumptions regarding future pregnancy risk, clinician lack of knowledge and comfort, and/or fragmentations of care with consultant or subspecialty care teams [10].

Permanent contraception remains one of the most utilized forms of contraception in the United States [11–14]. Given the multi-level factors and barriers to permanent contraception at the patient-, physician-, hospital-, and policy-level, prenatal counseling is hypothesized to improve postpartum receipt of desired permanent contraception [13]. For example, the federal Medicaid sterilization policy mandates a 30-day waiting period between the time that the required consent form is signed and when surgery can be completed [15, 16]. If prenatal contraceptive counseling is not performed, patients with Medicaid who desire permanent contraception face structural barriers to inpatient postpartum permanent contraception and must instead pursue interval outpatient permanent contraception with its own associated barriers to fulfillment [15, 17]. Secondly, the consistency, certainty, and length of desire regarding a patient’s goal for permanent contraception may impact physician counseling and practice [18, 19]. However, little data is available on the association between prenatal contraceptive counseling and fulfillment of postpartum permanent contraception.

While occurrence and quality of prenatal contraceptive counseling have been variably measured in prior research, the current study examines documentation of a plan for permanent contraception in the electronic medical record. Documentation of a plan for permanent contraception signified that contraceptive counseling occurred, that a plan was determined, and that the plan was documented to communicate to other members of the clinical team. We sought to investigate the association between initial timing of prenatal documentation of a plan for permanent contraception as the postpartum contraceptive method and fulfillment of postpartum permanent contraception. We hypothesized that patients with earlier trimester (trimester one or trimester two) of first documented plan for desired permanent contraception would have a higher likelihood of fulfillment before hospital discharge, within 42 days of delivery, and within 365 days of delivery compared to those with later trimester of first documented plan (trimester three). Additionally, we hypothesized that patients with a plan for permanent contraception documented during prenatal care would have higher odds of fulfillment at each timepoint than those patients whose plan for permanent contraception was first documented during the delivery hospitalization.

## Methods

We completed a multi-site retrospective cohort study of patients who delivered at or beyond 20 weeks gestation between January 1, 2018 and December 31, 2019. Four different hospitals participated in this study: University of California San Fransisco, Northwestern Memorial Hospital in Chicago, MetroHealth Medical System in Cleveland, and University of Alabama at Birmingham. Full study methodology of the primary study has been published [20]. Briefly, our study included patients who had permanent contraception documented as their contraception plan on the electronic medical record during their delivery hospitalization. Our exclusion criteria removed from the study patients who (i) suffered peripartum mortality; (ii) had previously received permanent contraception but were pregnant by in-vitro fertilization; (iii) had planned cesarean hysterectomy due to suspected placenta accreta spectrum; and (iv) had missing data across study variables. Data were abstracted from both inpatient and outpatient electronic medical records. The study was approved by the MetroHealth Medical Service institutional review board.

### Outcomes

The primary outcome for this study was fulfillment of permanent contraception recorded dichotomously at three timepoints: before discharge, within 42 days of delivery, or within 365 days of delivery.

### Key predictors

In this analysis, our primary exposure was the trimester (first, second, or third) during which permanent contraception was first documented as a patient’s desired method of postpartum contraception. If the plan for desired permanent contraception was documented in multiple trimesters, the earliest documentation was chosen for analysis. In addition, patients may have had other forms of contraception documented as their postpartum plan in addition to their plan for postpartum contraception or in previous trimesters before deciding on permanent contraception. General prenatal documentation was coded if timing of the prenatal documentation was not available in chart abstraction, but documentation was evident prenatally. If there was no prenatal documentation of a plan for permanent contraception but this decision was first documented during their delivery hospitalization, a patient was analyzed as having an initial documented plan for permanent contraception during their delivery hospitalization.

### Covariates

We prespecified all covariates prior to multivariable analysis. Covariates included insurance status (binary; Medicaid or non-Medicaid), maternal age at delivery (continuous), parity before delivery (binary; less than two or two or more), weeks of gestation at delivery (continuous), mode of delivery (binary; cesarean or vaginal), adequacy of prenatal care defined by the Kotelchuck Index [21] (binary; adequate or inadequate), maternal race (categorical; Black, Asian, White, other), ethnicity (categorical; Hispanic or non-Hispanic), marital status (binary; married or single), and body mass index (BMI) (continuous). Covariates were chosen as they are known to be associated with choice for and access to permanent contraception [22, 23]. Race and ethnicity were included as social constructs, recognizing ongoing evidence of racism in prenatal care delivery and historical evidence of reproductive coercion within historically marginalized communities that may impact both prenatal counseling and completion of permanent contraception procedures. [24]

### Statistical analysis

Using non-parametric t-tests for continuous variables and chi-squared for categorical variables, we compared our demographic and clinical variables across patients who had documented prenatal counseling for permanent contraception and those who did not. We then conducted univariate analysis as well as built a multivariable logistic regression model to examine the relationship between a first documented prenatal plan for permanent contraception in the first or second trimester and the odds of desired permanent contraception fulfillment at hospital discharge, six weeks (42 days), and one year (365 days) postpartum when compared to a first documented plan in the third trimester. We repeated this analysis investigating the relationship between a prenatal plan for permanent contraception at any timepoint versus documentation first occurring during the delivery hospitalization and the odds of fulfillment at each timepoint. To correct for any clustering across study sites, we estimated robust standard errors. Statistical significance was set with an α of 0.05. All statistical modeling was performed using R Version 4.2. [25]

## Results

Of the 49,915 deliveries across the four sites, 3013 patients met inclusion criteria and comprised our final analytical sample. 2,083 (69.1%) had prenatal documentation of desire for permanent contraception as their method of choice for postpartum contraception. Patients who had a documented plan for postpartum permanent contraception were younger, had higher parity prior to delivery, had received adequate prenatal care, were more likely to identify as Black, were less likely to identify as Hispanic, were unmarried, and had Medicaid insurance compared to patients who did not have a documented prenatal plan for permanent contraception (Table 1).

**Table 1:** Demographic and clinical characteristics of patients who desired postpartum permanent contraception by timing of documented plan for postpartum contraception, (*n* = 3013)

	Documented prenatal plan for permanent contraception	No documented prenatal plan for permanent contraception	*p*-value
Number of birthing people	2083	930	
Maternal age at delivery (years)	32 [28, 36]	33 [29, 37]	< 0.001
Parity less than 2 at admission	506 (24.3)	320 (33.3)	< 0.001
Weeks of gestation at delivery	38.5 [37.0, 39.1]	38.3 [36.5, 39.1]	0.027
Adequacy of prenatal care			< 0.001
Inadequate	524 (25.2)	173 (18.6)	
Intermediate	312 (15.0)	83 (8.9)	
Adequate	691 (33.2)	148 (15.9)	
Adequate plus	524 (25.2)	88 (9.5)	
Missing	32 (1.5)	438 (47.1)	
Delivery type			0.002
Caesarean section	967 (46.4)	488 (52.5)	
Vaginal delivery	1116 (53.6)	442 (47.5)	
Maternal race			< 0.001
Asian	61 (2.9)	28 (3.0)	
Black	940 (45.1)	304 (32.7)	
White	670 (32.2)	372 (40.0)	
None of the above	190 (9.1)	52 (5.6)	
Declined or unknown	222 (10.7)	174 (18.7)	
Maternal ethnicity non-Hispanic	1581 (75.9)	654 (70.3)	0.001
Married	688 (33.0)	437 (47.0)	< 0.001
BMI (kg/m^2^)	34.2 [29.6, 40.0]	33.2 [29.1, 38.4]	0.009
Insurance type private	521 (25.0)	416 (44.7)	< 0.001

Data represented as *n* (%) or median [interquartile range]

*BMI* body mass index

The relationship between timing of first documentation of the plan for permanent contraception and fulfillment of that plan is shown in Table 2. In univariable analysis, patients with a plan for permanent contraception in the first or second compared to third trimester were more likely to achieve fulfillment of desired permanent contraception prior to hospital discharge (OR 1.41, 95% CI 1.17–1.69), within 42 days of delivery (OR 1.37, 95% CI 1.14–1.65), and within 365 days  of delivery (OR 1.36, 95% CI 1.13–1.65). In our multivariable regression model, having a documented plan for permanent contraception in the first or second trimester, when compared to an initial documented plan in the third trimester, was associated with higher odds of fulfillment before discharge (aOR 1.57, 95% CI 1.24–2.00), 42 days (aOR 1.51, 95% CI 1.20–1.91), and 365 days (aOR 1.40, 95% CI 1.11–1.75) postpartum (Table 3).

**Table 2:** Timing of first documented plan for permanent contraception and fulfillment of permanent contraception at discharge, by 42 days, and by 365 days postpartum

	Trimester 1 (*n* = 523)	Trimester 2 (*n* = 721)	Trimester 3 (*n* = 757)	Any Prenatal (*n* = 82)	All Prenatal Combined (*n* = 2083)	Delivery Hospitalization (*n* = 930)
Fulfillment prior to discharge	324 (61.6)	447 (62.1)	406 (53.6)	57 (69.5)	1234 (59.2)	525 (56.5)
Fulfillment by 42 days	329 (62.9)	454 (62.9)	419 (55.4)	59 (72.0)	1261 (60.5)	530 (57.0)
Fulfillment by 1 year	362 (69.2)	512 (71.0)	480 (63.4)	62 (75.6)	1416 (67.7)	560 (60.2)

Presented as *n* (%)

**Table 3:** Multivariable logistic regression results of permanent contraception fulfillment by discharge, 42 days, and 365 days postpartum for patients with plan for permanent contraception documented in the first or second compared to third trimester (*n* = 2001)

	Hospital Discharge aOR (95% CI)	42 Days aOR (95% CI)	365 Days aOR (95% CI)
Documented prenatal plan for permanent contraception in first or second trimester^*^	1.57 (1.24–2.00)	1.51 (1.20–1.91)	1.40 (1.11–1.75)
Private insurance^†^	1.45 (1.07–1.97)	1.32 (0.98–1.79)	1.18 (0.88–1.59)
Maternal age at delivery^‡^	0.99 (0.97–1.97)	0.99 (0.97–1.02)	0.99 (0.96–1.01)
Parity ≥ 2^§^	1.15 (0.87–1.52)	1.08 (0.82–1.43)	1.02 (0.78–1.35)
Weeks of gestation at delivery^‡^	1.11 (1.06–1.16)	1.12 (1.07–1.17)	1.11 (1.06–1.17)
Cesarean section^||^	18.26 (14.03–23.76)	16.21 (12.50–21.03)	10.19 (7.86–13.21)
Adequate prenatal care^¶^	1.21 (0.96–1.52)	1.18 (0.94–1.48)	1.46 (1.17–1.83)
Race			
Asian^#^	0.60 (0.30–1.22)	0.64 (0.32–1.29)	0.92 (0.46–1.86
Black^#^	0.95 (0.72–1.25)	0.93 (0.73–1.22)	0.80 (0.61–1.04)
Hispanic Ethnicity^**^	0.87 (0.56–1.35)	0.83 (0.54–1.28)	1.25 (0.81–1.95)
Married^††^	1.29 (0.98 -1.71)	1.33 (1.01–1.75)	1.15 (0.87–1.51)
BMI^‡^	0.95 (0.94–0.97)	0.96 (0.94–0.97)	0.97 (0.95–0.98)

All variables shown in the table were included in the multivariable model

*OR*  odds ratio, *CI*  confidence interval

^*^Referent group—Documented prenatal plan for permanent contraception in third trimester

^†^Referent group—Medicaid insurance

^‡^Analyzed continuously

^§^Referent group—Parity < 2

^||^Referent group—Vaginal delivery

^¶^Referent group—Inadequate prenatal care

^#^Referent group—White; other and declined/unknown were also included in the model

^**^Referent group—Non-Hispanic

^††^Referent group—Unmarried

We also explored the relationship between prenatal documentation versus documentation first occurring during delivery hospitalization and fulfillment of permanent contraception. In univariable analysis, patients with prenatal documentation were not more likely to undergo permanent contraception prior to discharge (OR 1.12, 95% CI 0.96–1.31) or 42 days postpartum (OR 1.16, 95% CI 0.99–1.35) but were prior to 365 days postpartum (1.40, 95% CI 1.19–1.65). However, after multivariable analyses this relationship was significant at each time point (Table 4). Having a documented plan for permanent contraception at any prenatal timepoint was associated with higher odds of fulfillment before discharge (aOR 1.60, 95% C.I. 1.29–1.99), at 42 days (aOR 1.66, 95% C.I. 1.34–2.05), and 365 days (aOR 1.92, 95% C.I. 1.56–2.36) postpartum (Table 4).

**Table 4:** Multivariable logistic regression results of permanent contraception fulfillment by discharge, 42 days, and 365 days postpartum for patients with plan for permanent contraception documented prenatally compared to first during delivery hospitalization (*n* = 3013)

	Hospital Discharge	42 Days	365 Days
	Multivariable OR (95% CI)	Multivariable OR (95% CI)	Multivariable OR (95% CI)
Documented prenatal plan for permanent contraception^*^	1.60 (1.29–1.99)	1.66 (1.34–2.05)	1.92 (1.56–2.36)
Private insurance^†^	1.59 (1.25–2.01)	1.54 (1.22–1.95)	1.47 (1.16–1.86)
Maternal age at delivery^‡^	1.00 (0.98–1.02)	1.00 (0.98–1.02)	1.00 (0.98–1.50)
Parity ≥ 2^§^	1.36 (1.10–1.70)	1.31 (1.05–1.63)	1.21 (0.98–1.50)
Weeks of gestation at delivery^‡^	1.11 (1.08–1.15)	1.12 (1.08–1.15)	1.11 (1.07–1.14)
Cesarean section^||^	17.50 (14.12–21.67)	15.91 (12.86–19.66)	10.50 (8.52–12.94)
Adequate prenatal care^¶^	1.11 (0.91–1.34)	1.11 (0.92–1.34)	1.34 (1.11–1.61)
Race			
Asian^#^	0.51 (0.29–0.92)	0.52 (0.29–0.92)	0.63 (0.36–1.10)
Black^#^	1.01 (0.80–1.26)	0.97 (0.77–1.21)	0.81 (0.65–1.01)
Hispanic Ethnicity^**^	1.07 (0.77–1.47)	1.03 (0.74–1.42)	1.18 (0.86 -1.62)
Married^††^	1.43 (1.15 -1.78)	1.43 (1.15–1.78)	1.29 (1.04–1.60)
BMI^‡^	0.96 (0.95–0.97)	0.96 (0.95–0.97)	0.97 (0.96–0.98)

All variables shown in the table were included in the multivariable model

*OR*  odds ratio, *CI*  confidence interval

^*^Referent group—Plan for permanent contraception first documented during delivery hospitalization

^†^Referent group—Medicaid insurance

^‡^Analyzed continuously

^§^Referent group—Parity < 2

^||^Referent group—Vaginal delivery

^¶^Referent group—Inadequate prenatal care

^#^Referent group—White; other and declined/unknown were also included in the model

^**^Referent group—Non-Hispanic

^††^Referent group—Unmarried

## Discussion

In our multi-center retrospective cohort study, patients who had their first plan for permanent contraception documented in the first or second trimester had, at all-time points, approximately one and a half times higher odds of undergoing surgery for desired permanent contraception compared to patients whose first prenatal plan for permanent contraception was in the third trimester. This finding emphasizes the importance of initiating early prenatal counseling for permanent contraception. Furthermore, patients with any prenatal contraceptive plan for permanent contraception had a higher likelihood of fulfillment compared to patients who did not, as shown in the multivariable analyses. This aligns with prior studies demonstrating that patients who receive prenatal counseling are more likely to receive postpartum contraception in general [3–6]. Given the known multi-level barriers to postpartum permanent contraception, our findings suggest that early counseling is important to ensure that patients have time to discuss and finalize their contraceptive plan, that a plan for fulfillment can be made that mitigates known barriers such as operating room and surgeon availability, and that the required Medicaid sterilization consent form can be signed and necessary waiting period elapsed prior to delivery [16, 20].

We are not able to determine from this retrospective chart review study the reason behind this positive association. We hypothesize that this relationship may be multifactorial and likely related to patients strongly desiring permanent contraception initiating discussions early in pregnancy, patient desire for permanent contraception versus acceptance of alternative options impacting fulfillment, and clinician advocacy surrounding fulfillment of patient-centered contraceptive goals. This association could also be related to the role of implicit bias impacting counseling and care. For example, the association between early counseling and fulfillment persisted despite adjustment for adequacy of prenatal care. This is likely because the Kotelchuck Index, which is used to determine adequacy of prenatal care, does not measure the quality of prenatal care, but rather the number of visits [26]. This difference may reflect advocacy on the part of clinicians to ensure that health-related social needs and other barriers to outpatient care do not impinge on a patient’s goals for postpartum contraception.

Alternatively, the lack of association between adequacy of prenatal care and timing of prenatal plan for desired postpartum contraception may reflect implicit biases physicians have regarding contraceptive counseling and provision for those with structural and social barriers to care. Our study demonstrated racial and ethnic differences in rates of prenatal documentation of a plan for postpartum permanent contraception. Multiple studies have shown providers weave bias surrounding patients’ race, ethnicity, socioeconomic status, and age into contraception counseling for permanent contraception [18, 19, 27, 28]. The impact of such bias in counseling on rates of desire and fulfillment of permanent contraception is less clear with prior studies reporting both increased and decreased rates of use of permanent contraception by Black and Hispanic compared to White patients [29–31]. Regardless, ensuring providers acknowledge and mitigate their implicit biases is essential to upholding patient autonomy during prenatal contraceptive counseling. As the American College of Obstetricians and Gynecologists states, it is imperative for physicians to not deny postpartum contraception “because of physician ideals and values” regarding who is appropriate to receive this form of contraception [13]. This further underscores the importance of providers engaging in and documenting counseling with all pregnant patients regarding postpartum contraception early and throughout pregnancy. Such contraceptive counseling should be done supportively via shared decision-making within a reproductive justice framework [32].

Our study is potentially limited by lack of follow-up, given it is a retrospective cohort study. Additionally, we only were able to investigate when a patient had documentation of a plan for desired postpartum permanent contraception. Using documentation as a proxy for decision-making does not capture all patients who had counseling for contraception but did not specifically desire permanent contraception at a given time. This may have resulted in an underestimation of patients receiving prenatal counseling for postpartum contraception at each timepoint. Additionally, we were not able to assess or evaluate the occurrence of any postpartum contraceptive counseling that may have occurred after hospital discharge or the rationale for why surgery for permanent contraception was not performed. Future prospective study is needed to better delineate the quality of the contraceptive counseling, assess differences between patients (including potentially by factors such as race and ethnicity), document longitudinal patient decision-making and clinician counseling, and understand the rationale for non-fulfillment of permanent contraception.

## Conclusion

Our multi-site, diverse, national cohort study demonstrated that patients with documented desire for permanent contraception prenatally were more likely to have fulfillment after accounting for relevant clinical and demographic factors. Further, we show patients who have earlier documentation of a prenatal plan for permanent contraception (first or second trimester) had higher odds of fulfillment compared to patients who had documentation for a plan later in pregnancy (third trimester). Prenatal counseling and documentation surrounding permanent contraception may assist in mitigating the barriers associated with fulfillment of postpartum permanent contraception. This study highlights the necessity of counseling for postpartum contraception earlier in the prenatal period to allow for patients and clinicians to engage in longitudinal, shared decision-making.

## Data Availability

The dataset used and/or analyzed during the current study are available from the corresponding author on reasonable request.
